# Light-induced evaporative cooling of holes in the Hubbard model

**DOI:** 10.1038/s41467-019-13557-9

**Published:** 2019-12-05

**Authors:** Philipp Werner, Martin Eckstein, Markus Müller, Gil Refael

**Affiliations:** 1 Department of Physics, University of Fribourg, 1700 Fribourg, Switzerland; 2 Department of Physics, University of Erlangen-Nürnberg, 91058 Erlangen, Germany; 3 Paul Scherrer Institute, Condensed Matter Theory, PSI, Villigen, Switzerland; 4 Department of Physics, California Institute of Technology, Pasadena, CA 91125 USA

**Keywords:** Electronic properties and materials, Theoretical physics

## Abstract

An elusive goal in the field of driven quantum matter is the induction of long-range order. Here, we propose a mechanism based on light-induced evaporative cooling of holes in a correlated fermionic system. Since the entropy of a filled narrow band grows rapidly with hole doping, the isentropic transfer of holes from a doped Mott insulator to such a band results in a drop of temperature. Strongly correlated Fermi liquids and symmetry-broken states could thus be produced by dipolar excitations. Using nonequilibrium dynamical mean field theory, we show that suitably designed chirped pulses may realize this cooling effect. In particular, we demonstrate the emergence of antiferromagnetic order in a system which is initially in a weakly correlated state above the maximum Néel temperature. Our work suggests a general strategy for inducing strong correlation phenomena in periodically modulated atomic gases in optical lattices or light-driven materials.

## Abstract (web summary)

Driven quantum many-body systems can host finite densities of quasiparticles with the potential to realise emergent behaviour that is distinct from the equilibrium state. Werner et al. propose a method to cool holes in a correlated system so that more exotic low-entropy phases can be reached.

## Introduction

Inducing or enhancing electronic orders by some nonequilibrium process is an intriguing prospect, which captured the attention of many scientists^1–12^ following the recent observation of an apparent high-temperature superconducting state in light-driven cuprates^13,14^ and fulleride compounds^15^. Some of the theoretical proposals which have been put forward to explain these experiments focus on the cooling of quasi-particles or phase fluctuations in a periodically driven state. In ref. ^10^, it has been argued that the quasi-particles in phonon-driven K${}_{3}$C${}_{60}$ are cooled via the creation of entropy-rich spin-triplet excitons. In an earlier study focusing on bi-layer cuprates^1^, the authors showed that a time-periodic modulation of the plasma frequency may result in the cooling of the low-energy inter-bi-layer plasmon modes by energy transfer to the high-energy intra-bi-layer plasmon modes. While these proposals are very interesting, they rely so far on relatively simple model calculations which may not fully capture the interaction effects in driven many-body systems.

Simulations of photo-excited or phonon-driven correlated electron systems^4,17^ based on nonequilibrium dynamical mean field theory (DMFT)^16^ have generically produced heating effects and a melting of electronic orders^12,18,19^. Moreover, the formation of quasi-particles in photo-doped Mott insulators has been found to be extremely slow^20^, even in the presence of cooling by a phonon bath. While an enhancement of pairing susceptibilities in photo-doped Mott insulators has been reported^21^, the observed effect was too small to trigger a symmetry-breaking. So far, it thus remained unclear if quasi-particle cooling and symmetry-broken phases can be induced by periodic driving or photo-doping if heating and thermalization effects are accounted for.

Here, we demonstrate effective cooling by optically enabling the evaporation of holes from a system of interest (e.g., a partially filled Hubbard band) into a completely filled, almost flat band. In cold atom experiments the two subsystems could be realized by chains or layers at different potentials. In condensed matter, the full band represents a low-lying ligand band or core states. The specific protocol involves coupling a hole-doped Mott insulator by dipolar excitations to the initially filled narrow band. Since the entropy increase per hole is large in a full narrow band, a transfer of holes at constant total entropy results in strong cooling; a feature shared with other isentropic cooling schemes^11,22,23^. In the rotating frame, optical driving between the bands induces a tunneling from the Hubbard band to the flat band, whose energy is shifted by the driving frequency $\Omega$. The evaporative cooling is most effective if $\Omega$ matches the difference in effective chemical potentials between the two bands. In this case, hot holes are ejected from the Hubbard band in a narrow energy region below the chemical potential, which produces a steeper (and hence colder) distribution. The entropy of the system can be reduced by this mechanism down to a temperature which is essentially limited by the width of the narrow band. Using nonequilibrium DMFT simulations, we show that this idealized scenario can be approximately realized by simple and realistic driving protocols, resulting in optically induced cooling and long-range order.

## Results

### DMFT results for a large number of core bands

**Fig. 1:**
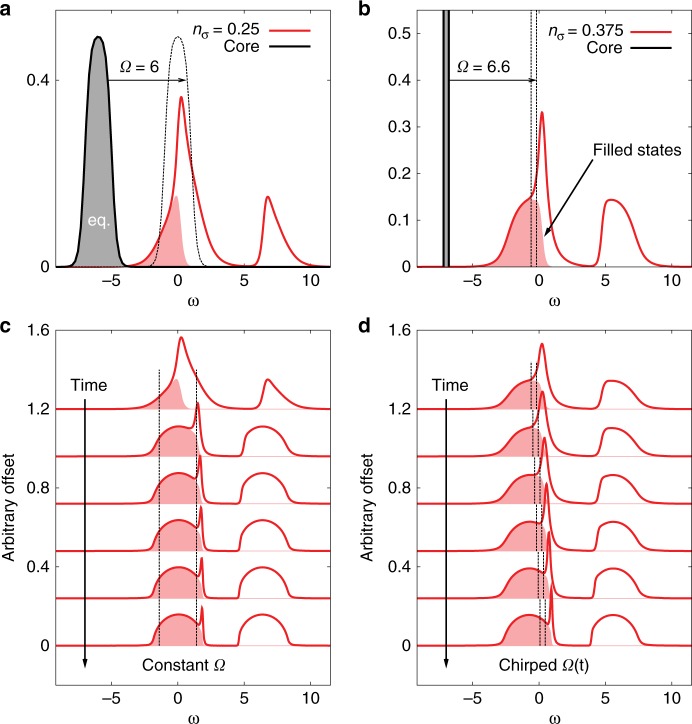
Spectral functions before and after photo-excitation. Panel **a** illustrates the initial state in the first setup: a quarter-filled Hubbard model (red) and core states of bandwidth 2 (black), which are treated as a noninteracting fermion bath. Panel **b** shows the second set-up: a 3/8 filled Hubbard model (red) and a narrow core band of width 0.4 (black). In both cases, the interaction parameters are ${U}_{{\rm{system}}}=6$, ${U}_{{\rm{core}}}=0$, and the initial temperature is $T=0.2$. Panel **c** illustrates the evolution of the spectral function (lines) and occupation (shaded region) with pulse duration in the first setup, for constant driving frequency $\Omega =6$ and amplitude ${a}_{\max }=0.8$. Panel **d** shows analogous results for the second set-up and a chirped pulse with ${\Omega }_{{\rm{in}}}=6.6$ and amplitude ${a}_{\max }=0.1625$.

We will consider two set-ups, representing the opposite limits of a large number of narrow bands, and a single narrow band, respectively. The first set-up (Fig. 1a, c) consists of a Hubbard model on an infinite-dimensional Bethe lattice, which can be solved exactly using DMFT^24^, and narrow bands represented by a noninteracting fermion bath, which remains in equilibrium at constant temperature and filling (see Methods). Henceforth, we refer to the Hubbard model as the system and to the full narrow band(s) as the core. The bandwidth of the noninteracting system is $4v$ (corresponding to a hopping ${v}_{{\rm{system}}}=v$), that of the core band is $2v$, and we use $v$ ($\hslash {v}^{-1}$) as the unit of energy (time). (For a bare bandwidth of 4 eV, the unit of time is 0.66 fs.) Particles are transferred from the filled noninteracting core levels (fermion bath) into the lower Hubbard band via a dipolar excitation with frequency $\Omega$, maximum amplitude ${a}_{\max }$ and pulse length $\delta$ (see Methods).

In Fig. 1a, we show the density of states (DOS) of the system (red) and core levels (black) in the initial state, together with their occupations (shaded regions). The Hubbard interaction $U=6$ is larger than the bandwidth, so that the DOS of the system is split into upper and lower Hubbard bands. We start in a quarter-filled state, with chemical potential in the lower Hubbard band. The dashed black line represents the core level DOS shifted by $\Omega =6$. The initial temperature of the system and core bands is $T=0.2$ (inverse temperature $\beta =5$), which is above the maximum ${T}_{{\rm{N}}\acute{\rm{e}}{\rm{el}}} \approx 0.15$ reached for $U=6$ at half-filling^18^.

**Fig. 2:**
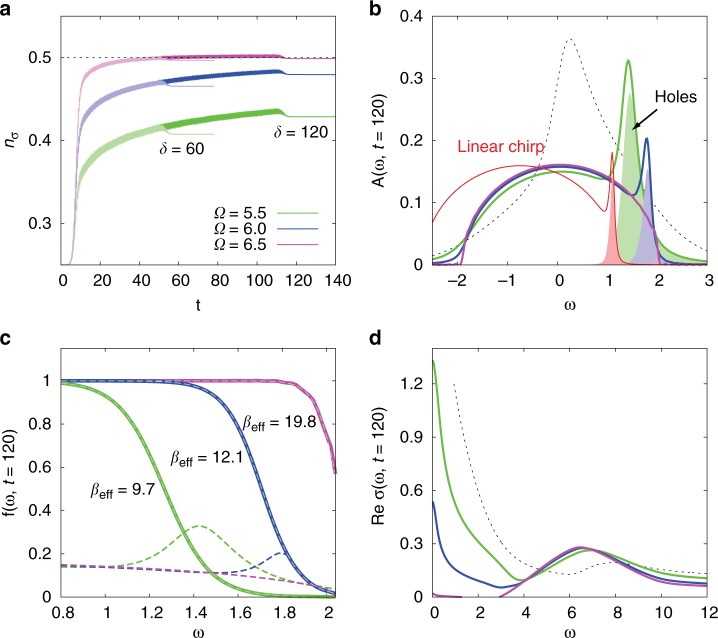
Photo-doping and quasi-particle cooling in the first set-up. Panel **a** Time evolution of the filling during a pulse with amplitude ${a}_{\max }=0.8$ and indicated frequencies $\Omega$ and pulse durations $\delta$. Panel **b** Spectral functions (lower Hubbard band) measured after the longer pulse. Shaded regions show the hole occupation. Panel **c**: Energy distribution function $f(\omega ,t)$ measured after the longer pulse. The dashed gray lines are Fermi functions corresponding to the indicated inverse temperatures ${\beta }_{{\rm{eff}}}$. Dashed colored lines show the spectral functions. Panel **d** Real part of the optical conductivity measured after the longer pulse. The dashed black lines in panels **b**, **d** show the result for the initial quarter-filled state at $\beta =5$. The pulse amplitude is ${a}_{\max }=0.8$. (The red spectrum in panel **b** shows the result for the second set-up and a chirped pulse of length $\delta =90$, amplitude $0.1625$, ${\Omega }_{{\rm{in}}}=6.6$ and ${\Omega }_{{\rm{fin}}}=7.4$).

The dipolar excitations in the chosen range of driving frequencies $\Omega$ lead to an increase in the occupation per spin ${n}_{\sigma }$ of the system. Figure 2a shows the time evolution of ${n}_{\sigma }$ for different pulse frequencies and fixed pulse amplitude ${a}_{\max }=0.8$. While the amplitude dependence is non-monotonous, longer pulses result in a larger increase of ${n}_{\sigma }$. The largest filling is obtained for ${a}_{\max }=0.8$ and $\Omega \approx 6.8$. In this case, a pulse of duration $\delta =120$ yields an almost complete filling of the lower Hubbard band (${n}_{\sigma }=0.499$), without any noteworthy increase in the number of doubly occupied sites. (Excitations into the upper Hubbard band by multiphoton absorption are possible in our set-up, but are strongly suppressed for the pulse amplitudes considered in this study.) Hence, the initially quarter-filled metallic system is switched to an (almost) Mott insulating state via the photo-induced particle transfer from the core levels. This is the opposite effect from the usual photo-doping^20,28–30^, which transforms a Mott insulator into a nonthermal metal via excitations across the Mott gap.

The light-induced metal-to-insulator transition is evident in the spectral function $A(\omega )$ and optical conductivity $\sigma (\omega )$, as illustrated in Fig. 2b, d, which show results measured immediately after the pulse of length $\delta =120$. The sharpness of the quasi-particle peaks in the spectral functions and of the Drude peaks in Re$\sigma (\omega )$ indicates a cold temperature of the photo-doped carriers. This is in stark contrast to the case of photo-doping across the Mott gap in a single-band Hubbard model, which typically results in hot charge carriers with a nonthermal distribution^28^ and very broad quasi-particle and Drude features^20^. To demonstrate the thermal nature of the photo-doped system and extract the corresponding temperature ${T}_{{\rm{eff}}}=1/{\beta }_{{\rm{eff}}}$ and chemical potential ${\mu }_{{\rm{eff}}}$, we plot in Fig. 2c the energy distribution functions $f(\omega ,t)$ measured after different pulses with $\delta =120$ (see Methods). The dashed gray lines indicate fits to a Fermi distribution ${f}_{F}(\omega ,{\mu }_{{\rm{eff}}},{\beta }_{{\rm{eff}}})=1/[1+\exp ({\beta }_{{\rm{eff}}}(\omega -{\mu }_{{\rm{eff}}}))]$, which very well match the measured distributions. We have confirmed that the equilibrium optical conductivities obtained for the measured ${n}_{\sigma }$ and ${\beta }_{{\rm{eff}}}$ reproduce the results shown in Fig. 2d. Hence, the Hubbard subsystem thermalizes rapidly after the decoupling from the core levels.

**Fig. 3:**
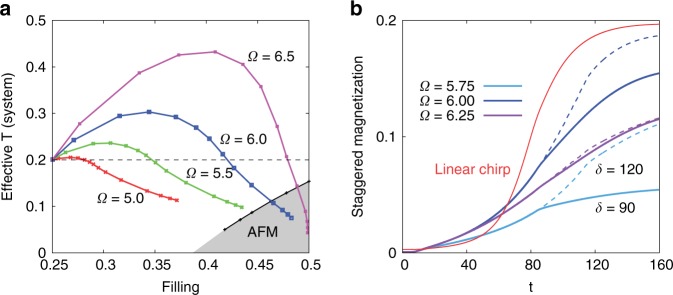
Photo-doping induced antiferromagnetic order in the first set-up. Panel **a** Effective system temperature after the pulse as a function of filling. The dashed line shows the temperature ($T=0.2$) of the initial quarter-filled state. Different curves correspond to different (but fixed) pulse frequencies $\Omega$, while different points correspond to different pulse durations $\delta$ (${a}_{\max }=0.8$). The black line shows the Néel temperature in equilibrium. Panel **b** Time dependence of the staggered magnetization for pulses with $\delta =90$ (solid lines) and 120 (dashed), ${a}_{\max }=0.8$ and indicated $\Omega$ in the presence of a staggered magnetic field $h=0.001$. (The red curve in panel **b** shows the result for the second set-up and a chirped pulse with parameters $\delta =90$, ${a}_{\max }=0.1625$, ${\Omega }_{{\rm{in}}}=6.6$ and ${\Omega }_{{\rm{fin}}}=7.4$).

Remarkably, the effective temperature of the photo-doped Hubbard model can be substantially lower than that of the initial equilibrium state ($T=0.2$). In Fig. 3a, we plot ${T}_{{\rm{eff}}}$ measured for different pulse frequencies and pulse durations as a function of the filling after the pulse. Long pulses ($\delta\, \gtrsim\, 90$) with $\Omega =6.5$ and ${a}_{\max }=0.8$ result in nearly half-filled systems with effective temperatures which are more than a factor of four lower than the initial temperature. ${T}_{{\rm{eff}}}$ can drop below the Néel temperature, which is indicated by the “AFM” line in Fig. 3a. Our results suggest that a state with antiferromagnetic long-range order can be realized in the present set-up by the combined effect of doping and cooling.

To demonstrate the possibility of inducing long-range antiferromagnetic order we apply a small staggered magnetic field $h=0.001$ to the system. The time evolution of the staggered magnetization $\langle {n}_{\uparrow }-{n}_{\downarrow }\rangle$ is plotted for different pulse frequencies $\Omega$ and pulse durations $\delta =90$ and $120$ in Fig. 3b (${a}_{\max }=0.8$). Already during the photo-excitation, a symmetry-breaking is induced, which increases further after the end of the pulse. The magnetization however saturates at a lower value than the effective temperatures and fillings in Fig. 3a would suggest, which is due to the heating of the system during the symmetry-breaking process. Also, we notice a slow-down of the dynamics close to half-filling, due to the suppressed hopping.

### DMFT results for a single core band

**Fig. 4:**
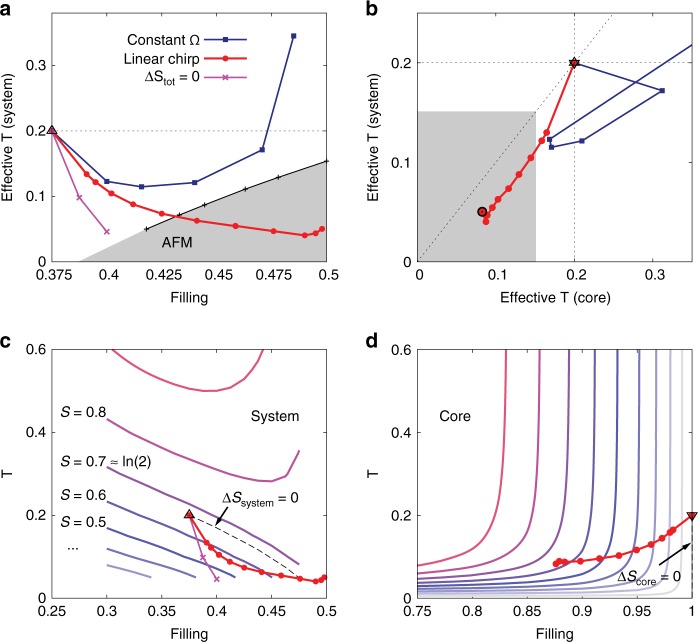
Cooling in the second set-up with a single narrow core band of width 0.4. Panel **a** Effective system temperature as a function of filling. The dark blue squares indicate the lowest temperatures reachable with constant $\Omega =6.8$, 7.0, 7.2, 7.4, 7.5 and optimized pulse amplitude ${a}_{\max }$ (from left to right), while the red dots are the results obtained for a chirped pulse with ${\Omega }_{{\rm{in}}}=6.6$, $6.7\le {\Omega }_{{\rm{fin}}}\le 7.4$ and optimized ${a}_{\max }$ (all data are for pulse length $\delta =90$). Pink crosses show the temperatures achievable with an insentropic charge transfer. Symmetry breaking to an antiferromagnetic state is expeced in the gray region. Panel **b** Effective system temperature against the effective core temperature for the same driving protocols as in panel **a**. With chirped pulses, both the system and core electrons are cooled below the initial temperature of 0.2. The gray shading indicates the Néel temperature of the system for the filling realized with the highest frequency pulse (black circle). Panel **c** The isentropy curves of the system in the space of filling and temperature, together with the data for the chirped pulse and the isentropic charge transfer. Both protocols lead to a decrase of the system entropy ${S}_{{\rm{system}}}$ over a large doping range. Panel **d** plots the isentropy curves of the core and the filling-versus-temperature data corresponding to the chirped pulse. The hole doping of the core band leads to a strong increase in the entropy ${S}_{{\rm{core}}}$. The up (down) triangles in panels **a**–**d** represent the initial state of the system (core), before the photo-doping.

Next, let us consider the second set-up in which the (noninteracting) and very narrow core band is equipped with a Bethe-lattice self-consistency (see Methods). To be able to reach fillings near ${n}_{\sigma }=0.5$, where the AFM order is most stable, we start from ${n}_{\sigma }=0.375$, as illustrated in Fig. 1b (${v}_{{\rm{system}}}=1$, ${v}_{{\rm{core}}}=0.1$). The dark blue squares in Fig. 4a show the lowest ${T}_{{\rm{eff}}}$ of the system realizable with the pulse parameters $\delta =90$, $\Omega =6.8,7.0,7.2,7.4,7.5$ (from left to right) and optimized amplitude ${a}_{\max }$. These results demonstrate that the cooling-by-doping mechanism also works if we couple to a single core band, although the Néel temperature cannot be reached with $\delta =90$.

Photo-excitation with a low $\Omega$ creates steep (and hence cold) distribution functions $f(\omega ,t)$ by filling high-kinetic energy holes in the correlated system with electrons from the core band, analogous to evaporative cooling, but the change in density is limited. In order to create a strongly correlated Fermi liquid with low temperature, we apply a chirped pulse of the form $a(t)={a}_{\max }\sin [\Omega (t)t]$ with $\Omega (t)$ increasing linearly from ${\Omega }_{{\rm{in}}}$ to ${\Omega }_{{\rm{fin}}}$ during the pulse of duration $\delta$. The results for $\delta =90$, ${\Omega }_{{\rm{in}}}=6.6$ and $0.67\le {\Omega }_{{\rm{fin}}}\le 7.4$ are shown by the red dots in Fig. 4. Here we again adjusted the amplitude ${a}_{\max }$ to realize the lowest ${T}_{{\rm{eff}}}$ of the system for each ${\Omega }_{{\rm{fin}}}$. (The lowest temperature of ${T}_{{\rm{eff}}}=0.04$ is reached for ${\Omega }_{{\rm{fin}}}=7.4$ and ${a}_{\max }=0.1625$; the gradual filling of the band with this type of pulse is illustrated in Fig. 1d.) With chirped pulses, we can easily access the strongly correlated Fermi liquid regime and trigger a symmetry breaking to the AFM phase, see Fig. 4a. The spectral function obtained after the ${\Omega }_{{\rm{fin}}}=7.4$, ${a}_{\max }=0.1625$ pulse is shown by the red curve in Fig. 2b, and the magnetization dynamics in the presence of a staggered field $h=0.001$ by the red curve in Fig. 3b.

### Cooling mechanism

The cooling mechanism can be understood by considering the entropy transfer between the two bands: While the entropy in the core band is initially zero, it strongly increases with hole-doping. If the population transfer could be achieved at fixed total entropy, one could thus decrease the system entropy and temperature. In practice, it is hard to realize an isentropic population transfer, but we will now show that suitable chirped pulses can nevertheless reduce the entropy of the system and that the corresponding reduction in temperature is enhanced by the fact that the system is driven towards the correlated Mott regime. For the quantitative analysis we compute the entropy per site $S$ of the noninteracting core band and of the Hubbard model as a function of filling and temperature $T$. Figure 4d shows the contour lines of the entropy for the core band, which we obtained numerically by integrating $C/T$ ($C$ is the specific heat per site) from $T=0$. The isentropic lines of the core band are almost vertical for temperatures above a scale set by its narrow bandwidth, corresponding to the infinite-temperature result ${S}_{\infty }=-2{n}_{\sigma }\;{\mathrm{ln}}({n}_{\sigma })-2(1-{n}_{\sigma })\;{\mathrm{ln}}(1-{n}_{\sigma })$. In the regime of interest to our simulation, the entropy increase of the core band is therefore approximately given by ${S}_{\infty }$. This is confirmed by the red circles, which show the effective core temperatures and fillings realized by the different chirped pulses. The entropy of the correlated ($U=6$) valence band is more interesting and is plotted in Fig. 4c. To obtain these data, we integrated $C/T$ from $T=\infty$. At low temperature, in a Fermi liquid, we expect $S(T)=\gamma T$ with $\gamma =\lim_{T\to 0}C/T$. In the doped Mott regime, the $\gamma$-factor diverges like $\gamma \propto 1/| 0.5-{n}_{\sigma }|$ near half-filling^31^, while the Fermi liquid coherence temperature (and hence the $T$-range for which ${S}_{{\rm{system}}}(T)=\gamma T$ holds) drops. In the paramagnetic insulator at ${n}_{\sigma }=0.5$, the entropy per site is roughly given by the remaining spin entropy $S=\mathrm{ln}(2)$ down to the lowest temperature, while states contributing to entropy $S\ > \ \mathrm{ln}(2)$ are only activated by charge fluctuations at temperatures of the order of $U$. This explains why the isentropic curves for $S\ \lesssim\ \mathrm{ln}(2)$ decrease toward zero near ${n}_{\sigma }=0.5$ while they increase with density for $S\ \gtrsim\ \mathrm{ln}(2)$, leading to a non-monotonous behavior of the isentropy curves as a function of filling for intermediate entropies.

An isentropic photo-doping process, in which the increase $\Delta {S}_{{\rm{core}}}\approx \Delta {S}_{\infty }\;> \; 0$ of the core entropy is compensated by a corresponding decrease of the system entropy, leads to a drastic reduction of the temperature as a function of the transferred charge (see magenta crosses in Fig. 4a, c). While a realistic process will never conserve the total entropy, the slope of the isentropy lines close to the Mott regime implies that even if the system entropy remained constant or increased slightly during a photo-doping process starting from the Fermi liquid regime, the system temperature can still decrease (see dashed line in panel c, which shows the line corresponding to $\Delta {S}_{{\rm{system}}}=0$). As indicated by the red dots, for the optimized chirped pulses, an actual decrease of the system entropy can be realized in a sizeable doping range, which implies a much stronger cooling than in the $\Delta {S}_{{\rm{system}}}=0$ case. This reshuffling of entropy from the system to the core band is the main mechanism behind the observed cooling effect.

The isentropy curves in panel c also explain why the photo-doping of the cold, half-filled Mott insulator generically results in strong heating: Here one starts with a large entropy of $\mathrm{ln}(2)$ per site, which is much larger than the entropy of a cold, Fermi-liquid-like metal. Hence, assuming that the entropy of a photo-doped insulator is similar to that of a chemically doped insulator, we conclude that even in the isentropic case, ${T}_{{\rm{eff}}}$ of the photo-doped state must strongly increase.

It is important though to note that the large entropy of the paramagnetic Mott insulator is a consequence of the single-site DMFT approximation. Nonlocal correlations can lead to singlet formation and reduce the entropy. Depending on the lattice structure and approximation used, the slopes of the isentropic lines in Fig. 4c can change, and hence also the optimal doping protocol may change. For example, the strongest cooling effect could be achieved away from half-filling, in which case it could be more effective to excite electrons into an empty narrow band, rather than holes into a full core band. What will not change is the primary cooling mechanism based on the reshuffling of entropy, and since our protocol has demonstrated a reduction of the temperature substantially below the antiferromagnetic transition in DMFT, we believe that the ordered phase can still be induced in the presence of nonlocal correlations.

The width of the core band matters for the cooling because it sets the temperature scale where the isentropic curves of the core bend from vertical to horizontal (see Fig. 4d). For a wider band, the bending occurs at higher temperature, which means that at fixed low temperature, the entropy increase with hole doping is reduced. We also note that both the system and core can be cooled (Fig. 4b). After the optimized chirped pulses their effective temperatures are similar, and both can be substantially below the Néel temperature of the system for the corresponding filling. While interband scattering processes would result in a synchronization of the effective temperatures, they would hence not dramatically change the observed cooling behavior.

## Discussion

Our simulations show that the particle transfer induced by dipolar excitations between a filled core band and a partially filled Hubbard band can be accomplished in such a way that entropy is shifted to the core band. It is interesting to compare this evaporative hole cooling mechanism to the established technique of thermal spin mixing^32^, used to efficiently cool electronic spins. There, a narrow band is formed by spin excitations that propagate via dipolar spin flip-flop terms^33–35^. By driving such excitations off the band center, and letting them relax with a phonon-bath, a steady state with an effective temperature of the order of the spin bandwidth is created. The evaporative cooling instead achieves low temperatures by using the narrow band as an efficient entropy sink. It could be interesting to explore combinations of the two mechanisms to optimize cooling protocols.

The set-ups explored in the present study provide a means to realize complex phases such as correlated Fermi liquids, antiferromagnetic, superconducting or excitonic order by the combined effect of doping and cooling^36^. The basic strategy is applicable to cold atom and condensed matter systems. In cold atom experiments, the two subsystems could be realized in separate layers, which are transiently coupled by a periodic hopping modulation or a shift in the local energies and tunnel barriers. It is also important to emphasize that our cooling mechanism works equally well if particles are shifted from the system to empty narrow bands, rather than out of full core bands. The bandstructure of the brickwall lattice^37^ features such a flat band. In fact, this lattice has been mainly implemented because the flat band allows to suppress unwanted excitations. In view of our results it would be interesting to explore resonant excitations into this flat band. Since the threshold entropy for antiferromagnetic order has recently been reached^38^, the additional cooling provided by the particle transfer could give access to the sought-after pseudo-gap and superconducting phases. In correlated materials, it is important to identify suitable (full or empty) flat bands, and to exploit the asymmetry in the laser excitations into and out of the bands to realize the reshuffling of entropy.

## Methods

### DMFT formalism

We use nonequilibrium DMFT^16^ to simulate the charge transfer from the core band to the system via dipolar excitations. The system is described by the Hubbard model ${H}_{{\rm{system}}}(t)=\tilde{v}(t){\sum }_{\langle i,j\rangle \sigma }({c}_{i\sigma }^{\dagger }{c}_{j\sigma }+{\rm{h.c.}})+U{\sum }_{i}{n}_{i\uparrow }{n}_{i\downarrow }-\mu {\sum }_{i}({n}_{i\uparrow }+{n}_{i\downarrow })$ on an infinitely connected Bethe lattice. Here, ${c}_{i\sigma }^{\dagger }$ creates a fermion on site $i$ with spin $\sigma$, $U$ is the Hubbard repulsion, $\tilde{v}$ the hopping between nearest neighbor sites, and $\mu$ the chemical potential. In DMFT^24^ this lattice model is mapped onto a quantum impurity model with action ${S}_{{\rm{imp}}}[U,{\Delta }_{{\rm{system}}}]$ defined by the on-site interaction $U$ and a hybridization function ${\Delta }_{{\rm{system}}}$. The latter is determined self-consistently in such a way that the Green function of the impurity model reproduces the local Green function of the lattice model^16,24^: ${G}_{{\rm{system}}}(t,t^{\prime} )\equiv -i{\rm{Tr}}[{T}_{{\mathcal{C}}}{e}^{{S}_{{\rm{imp}}}}c(t){c}^{\dagger }(t^{\prime} )]/{\rm{Tr}}[{T}_{{\mathcal{C}}}{e}^{{S}_{{\rm{imp}}}}]$. To evaluate the right hand side for a given ${\Delta }_{{\rm{system}}}$, we use the non-crossing approximation (NCA)^25,26^. The DMFT solution (with an exact impurity solver) becomes exact in the limit of infinite coordination number $z$, if the hopping is rescaled as ${v}_{{\rm{system}}}=\tilde{v}/\sqrt{z}$. In the case of the Bethe lattice the self-consistency condition for the model without core bands simplifies to ${\Delta }_{{\rm{system}},\sigma }(t,t^{\prime} )={v}_{{\rm{system}}}(t){G}_{{\rm{system}},\sigma }(t,t^{\prime} ){v}_{{\rm{system}}}(t^{\prime} )$.

AFM order can be studied in DMFT by considering two sublattices with opposite spin polarization and flipping the spin index of the hybridization function in the self-consistency equation^24^.

### Self-consistency for a large number of core bands

In the first set-up, the effect of the core bands is described by an additional hybridization function ${\Delta }_{{\rm{core}}}(t,t^{\prime} )={v}_{{\rm{system-core}}}(t){G}_{{\rm{core}}}^{0}(t,t^{\prime} ){v}_{{\rm{system-core}}}^{* }(t^{\prime} )$, where ${G}_{{\rm{core}}}^{0}$ is the Green function associated with the DOS of the core band. Since the core bands remain in equilibrium (i.e., fully filled), there is only one self-consistency equation for the hybridization function of the system

1 $${\Delta }_{{\rm{system}},\sigma }(t,t^{\prime} )=	\ {v}_{{\rm{system}}}(t){G}_{{\rm{system}},\sigma }(t,t^{\prime} ){v}_{{\rm{system}}}^{* }(t^{\prime} )\\ 	+{v}_{{\rm{system-core}}}(t){G}_{{\rm{core}}}^{0}(t,t^{\prime} ){v}_{{\rm{system-core}}}^{* }(t^{\prime} ).$$

Dipolar excitations from the core to the system are described by a time-periodic modulation ${v}_{{\rm{system-core}}}(t)=a(t)f(t-{t}_{p})$, where $a(t)={a}_{\max }\sin (\Omega t)$ is an oscillating function with frequency $\Omega$ and amplitude ${a}_{\max }$ and $f(t)=1/[(1+\exp (t-\delta +6))(1+\exp (-t+6))]$ a pulse envelope function of width $\delta$, with a ramp on and off time of approximately $2\times 6$. For $\Omega$ comparable to the energy difference between the core levels and the lower Hubbard band, this photo-excitation results in a transfer of charge from the core levels to the system. We describe the core levels by a box-shaped DOS with smooth edges in the energy range $-7\le \omega \le -5$ and consider driving frequencies $5\le \Omega \le 7$ and pulses of duration $\delta \approx 30\!-\!120$. For the pulse amplitudes ${a}_{\max }$ considered in this study, these multi-cycle pulses produce only a negligible number of double occupations, i.e., the upper Hubbard band remains empty.

### Self-consistency for a single-core band

In the second set-up, we describe the single core band by a noninteracting Hubbard model ($U=0$) of bandwidth 0.4, which like the system is solved with DMFT+NCA (to enable equilibration of the core electrons). In this case, the core band adds a hybridization term to the system’s impurity model and vice versa. The two coupled DMFT self-consistency equations become

2 $${\Delta }_{{\rm{system}},\sigma }(t,t^{\prime} )=	 \ {v}_{{\rm{system}}}(t){G}_{{\rm{system}},\sigma }(t,t^{\prime} ){v}_{{\rm{system}}}(t^{\prime} )\\ 	+{v}_{{\rm{system-core}}}(t){G}_{{\rm{core}},\sigma }(t,t^{\prime} ){v}_{{\rm{system-core}}}^{* }(t^{\prime} ),$$

3 $${\Delta }_{{\rm{core}},\sigma }(t,t^{\prime} )=	\ {v}_{{\rm{core}}}(t){G}_{{\rm{core}},\sigma }(t,t^{\prime} ){v}_{{\rm{core}}}(t^{\prime} )\\ 	+{v}_{{\rm{system-core}}}(t){G}_{{\rm{system}},\sigma }(t,t^{\prime} ){v}_{{\rm{system-core}}}^{* }(t^{\prime} ).$$

The effect of the periodic driving of ${v}_{{\rm{system-core}}}$ amounts to a shift of the core level DOS by $\Omega$ (see dashed lines in Fig. 1a, c). We have also performed simulations in which the core levels (and their chemical potential) are shifted by the energy $\epsilon =\Omega$ and the hopping between the core levels and the system is smoothly switched on and off. The results are qualitatively similar to those reported in the main text.

### Optical conductivity and spectral functions

This study does not consider direct excitations of the system by the pulse field. We have in mind a set-up where the hoppings within the system are perpendicular to the direction of the excitation pulse, while local dipole transitions are possible due to the opposite parity of the system and core orbitals. A Peierls phase factor in ${v}_{{\rm{system}}}(t)$ associated with a weak probe pulse parallel to the hopping is however added in order to measure the optical conductivity $\sigma$ of the system^16^. (For the implementation of the electric field in the Bethe lattice see ref. ^27^.) We apply a short electric field pulse ${E}_{t}(t^{\prime} )$ centered at a time $t=\delta$, i.e., immediately after the photo-doping pulse, and measure the induced current ${j}_{t}(t^{\prime} )$. After Fourier transformation, one obtains $\sigma (\omega ,t)={j}_{t}(\omega )/{E}_{t}(\omega )$. The time-dependent spectral function $A(\omega ,t)$ is calculated by forward integration of the retarded Green function, $A(\omega ,t)=-\frac{1}{\pi }{\rm{Im}}{\int }_{t}^{{t}_{\max }}dt^{\prime} {e}^{i\omega (t^{\prime} -t)}{G}^{{\rm{ret}}}(t^{\prime} ,t)$, and similarly the time-dependent occupation function ${A}^{<}(\omega ,t)$ is calculated from the lesser component^16^. The ratio $f(\omega ,t)={A}^{<}(\omega ,t)/{A}^{{\rm{ret}}}(\omega ,t)$ defines the time-dependent distribution function, which in a thermalized state becomes a Fermi distribution function.

## Data Availability

The data that support the findings of this study are available from the corresponding author upon reasonable request.

## Code availability

The codes used to produce the results of this study are based on a proprietary software library, which will be documented and published in the future.
